# Crystal structure of tris[(4,7,13,16,21,24-hexa­oxa-1,10-di­aza­bicyclo­[8.8.8]hexa­cosane-κ^8^ *N*_2_,*O*_6_)rubidium] rubidium nona­stannide

**DOI:** 10.1107/S2056989017000172

**Published:** 2017-01-13

**Authors:** Wilhelm Klein, Haiyan He, Thomas F. Fässler

**Affiliations:** a Technische Universität München, Department of Chemistry, Lichtenbergstr. 4, 85747 Garching, Germany

**Keywords:** tin, Zintl anions, cage compounds, stannides, crystal structure, isotypism

## Abstract

The crystal structure of the title compound, [Rb(C_18_H_36_N_2_O_6_)]_3_RbSn_9_, consists of deltahedral [Sn_9_]^4–^ cluster anions, Rb^+^ cations and cryptand mol­ecules, which partially sequester the cations. Those cations, which are not coordinated by cryptand mol­ecules, are neighboured directly to the [Sn_9_]^4–^ clusters and inter­connect them to form _∞_^1^[RbSn_9_]^3–^ chains. These chains extend parallel to [001] and are arranged in a pseudo-hexa­gonal rod packing, separated by the Rb-cryptand complex cations.

## Abstract 2

The title compound, [Rb(2.2.2)-crypt]_3_RbSn_9_, contains Rb^+^ cations, partially coordinated by 2.2.2-cryptand mol­ecules, and deltahedral nine-atomic tin cluster anions. The free Rb^+^ cations and the [Sn_9_]^4–^ anions form strands extending parallel to [001].

## Chemical context   

The dissolution of elemental tin in alkali metal ammonia solutions was reported first by Joannis (1891 ▸). Since then Zintl compounds containing tetrel elements, particularly the remarkably stable nine-membered cluster compounds, have been studied intensively. Plenty of chemical reactions such as reduction, oligomerization, functionalization, and even filling of the nine-membered clusters with transition metal atoms, have been investigated (Scharfe *et al.*, 2011 ▸). For enabling this extended variety of chemical reactions, dissolution of solid Zintl cluster compounds in organic solvents is often helpful or even necessary. To achieve this, the addition of sequestering agents like crown ethers or cryptands has been successfully applied (Corbett & Edwards, 1975 ▸). During our experiments including the Zintl cluster compound Rb_4_Sn_9_ in ethyl­enedi­amine in the presence of 2.2.2-cryptand, single crystals of the title compound, [Rb(2.2.2)-crypt]_3_RbSn_9_, have been obtained.
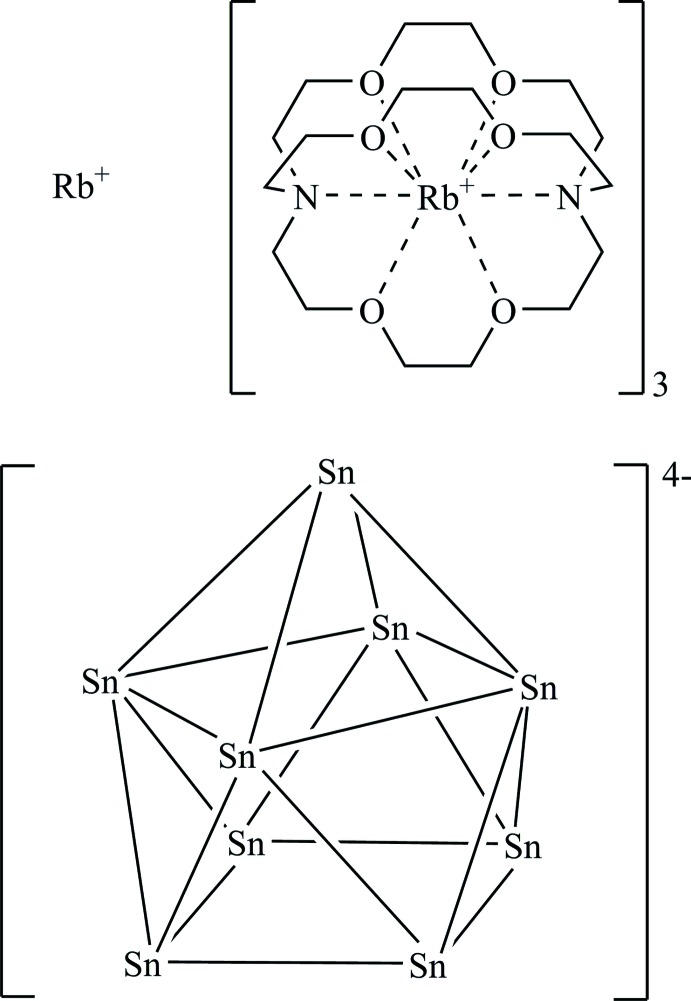


## Structural commentary   

**Figure 1:**
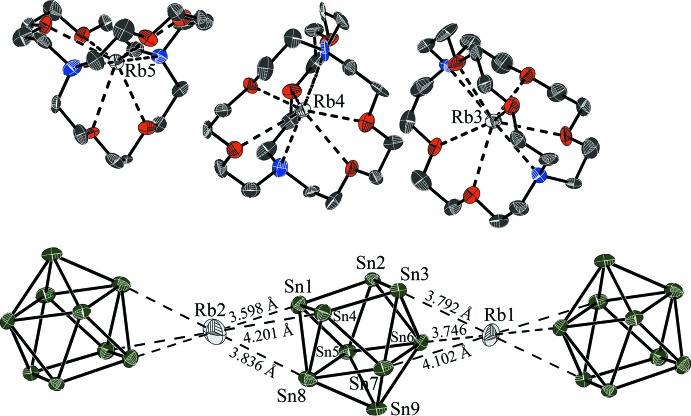
The main structural components of the title compound, showing a section of the _∞_^1^[RbSn_9_]^3−^ chain. Anisotropic displacement ellipsoids are drawn at the 50% probability level. H atoms of the cryptand mol­ecules have been omitted; labelled sections represent the asymmetric unit.

The title compound crystallizes in space group *P* 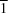 with all atoms at general sites except for Rb1 and Rb2, which are located on inversion centres (Wyckoff sites 1*a* and 1*b*). The crystal structure consists of five rubidium cations, partially sequestered by cryptand-[2.2.2], and nine-atomic Sn clusters, see Fig. 1 ▸. The composition of four Rb^+^ cations per Sn_9_ cluster anion indicate a fourfold negative charge and, thus, 22 skeleton electrons for [Sn_9_]. According to the Wade–Mingos electron-counting rules, the shape of such a [Sn_9_]^4–^ anion is predicted to be a *nido* cage, a mono-capped square anti­prism with *C*_4*v*_ symmetry (Fässler, 2001 ▸). Indeed, the cluster Sn atoms form an almost *C*_4*v*_ symmetric mono-capped square anti­prism, as indicated *e.g.* by the planarity and equal diagonal lengths of the square formed by atoms Sn1, Sn2, Sn3, and Sn4 [ratio of diagonals 1.03, dihedral angle between the two triangle halves of the square 3.59 (3)°]. However, larger devi­ations from the ideal *C*_4*v*_ symmetry are frequent, *e.g*. in the closely related compound [K(2.2.2)-crypt][K(18-crown-6)]_2_KSn_9_ (He *et al.*, 2014*a* ▸) where the [Sn_9_]^4–^ cluster exhibits a shape close to *D*_3*h*_ symmetry. The Sn_9_ clusters are capped by two crystallographically independent rubidium cations (Rb1 and Rb2) with Sn—Rb distances in the range between 3.5976 (10) Å and 3.8357 (10) Å; for both cations a third longer distance of more than 4 Å indicates an inter­mediate between edge-coordination and face-coordination at two opposite sites (Fig. 1 ▸). Both the cations are located at special crystallographic sites, Rb1 at 1*a* and Rb2 at 1*b*, so their surroundings, although irregular, are centrosymmetric. The title compound represents the fourth member of this structure type; however, it is the first one to contain Rb. The same type of ion packing has been found in the isotypic crystal structures of [K(2.2.2)-crypt]_3_KSn_9_ (Burns & Corbett, 1985 ▸), [K(2.2.2)-crypt]_3_K[Co_0.68_@Sn_9_] (He *et al.*, 2014*b* ▸), and [K(2.2.2)-crypt]_3_K[Ni@Sn_9_] (Gillett-Kunnath *et al.*, 2011 ▸). This structure type includes both empty Sn_9_ clusters and Sn_9_ clusters partly or completely filled with Co and Ni, respectively. As expected, the Sn—Sn bond lengths of the title compound are shorter than those in the filled cluster compounds. However, they are even slightly shorter than those of the empty [Sn_9_]^4–^ clusters in the potassium analogue [K(2.2.2)-crypt]_3_KSn_9_. A similar effect, namely decreasing homoatomic bond lengths with increasing size of the counter-cations, has been found for other homoatomic anions, *e.g*. [Sn_4_]^4–^ (Baitinger *et al.*, 1999*a* ▸,*b* ▸) and [O_3_]^−^ (Klein & Jansen, 2000 ▸). In the present case, this effect compensates the increase of inter­atomic distances resulting from the larger ionic radius of Rb^+^ compared to that of K^+^, so the unit-cell volume does even decrease slightly from 4186 Å^3^ (K^+^; Burns & Corbett, 1985 ▸) to 4166 Å^3^ (Rb^+^; title compound).

## Supra­molecular features   

**Figure 2:**
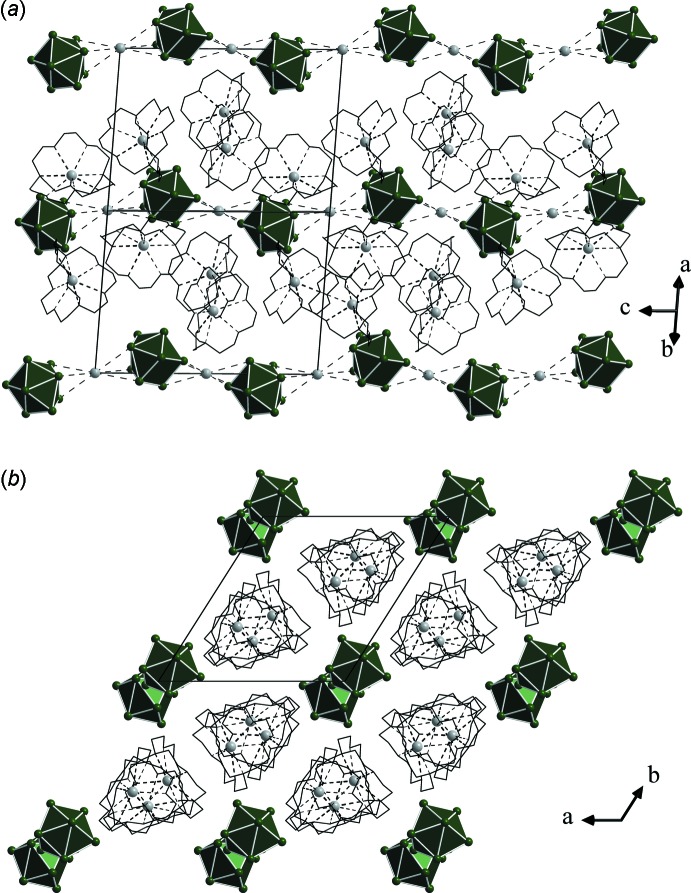
Crystal structure of the title compound: (*a*) in a view along [110]; (*b*) in a view along [001]. [Sn_9_]^4–^ clusters are drawn as polyhedra and Rb^+^ cations as grey spheres. H atoms of the cryptand mol­ecules have been omitted for clarity.

The [Sn_9_]^4–^ cluster anions are linked by rubidium cations formally into infinite _∞_^1^[RbSn_9_]^3−^ chains parallel to [001]. The shortest distances between Rb^+^ cations and Sn_9_ clusters are similar to those for related compounds, *e.g*. [Rb(18-crown-6)]_2_Rb_2_Sn_9_·1.5C_2_N_2_H_8_ (3.679 Å; Hauptmann & Fässler, 2002 ▸), [Rb(2.2.2)-crypt][Rb(18-crown-6)]Rb_2_Sn_9_·2NH_3_ (3.708 Å; Gaertner & Korber, 2011 ▸) or Rb_12_Sn_17_ (3.582 Å; Hoch *et al.*, 2003 ▸). The dimensionality of structural entities is influenced by the ratio of sequestered to unsequestered cations, and the formation of one-dimensional strands in Zintl phases of nine-atomic clusters with three sequestered cations has been observed previously (Fässler & Hoffmann, 1999 ▸; He *et al.*, 2014*a* ▸), while a lower content of sequestering agents promotes the formation of double strands (Gaertner & Korber, 2011 ▸), or of a layered arrangement (Hauptmann & Fässler, 2002 ▸, 2003*a* ▸,*b* ▸) or double layers (Hauptmann *et al.*, 2001 ▸). A complete coordination of all the cations finally leads to isolated clusters without direct contacts between clusters and cations. In the title compound, the chains form a pseudo-hexa­gonal rod packing separated by the [Rb(2.2.2)-crypt]^+^ complexes, as can be seen in Fig. 2 ▸ *b*.

## Database survey   

Nine-atomic Zintl cluster compounds and their chemistry have been reviewed by Fässler (2001 ▸) and by Scharfe *et al.* (2011 ▸). Rb_4_Sn_9_ has been crystallized in the presence of sequestering agents previously by Hauptmann & Fässler (2002 ▸) and Gaertner & Korber (2011 ▸). Binary phases of the elements Rb and Sn are known as the Zintl phases including [Sn_4_]^4–^ clusters, Rb_4_Sn_4_ (Baitinger *et al.*, 1999*b* ▸) and Rb_12_Sn_17_ (Hoch *et al.*, 2003 ▸), as well as the clathrate Rb_8_Sn_44_ (Dubois & Fässler, 2005 ▸). The structure type of the title compound has been reported previously by Burns & Corbett (1985 ▸), Gillett-Kunnath *et al.* (2011 ▸) and He *et al.* (2014*b* ▸).

## Synthesis and crystallization   

All manipulations were carried out under anhydrous and oxygen-free conditions using a glove-box or a Schlenk line. Ethyl­enedi­amine (Alfa–Aesar, 99%) and toluene were distilled over CaH_2_ and stored in a gas-tight Schlenk tube. Cryptand-[2.2.2] (4,7,13,16,21,24-hexa­oxa-1,10-di­aza­bicyclo­[8.8.8]-hexa­cosane, Acros, 98%) was dried under vacuum for 8 h. Rb_4_Sn_9_ was obtained from a stoichiometric mixture of the elements in a steel container, which was held at 823 K for 3 d under argon in a corundum tube. Rb_4_Sn_9_ (65 mg, 0.046 mmol) and cryptand-[2.2.2] (50 mg, 0.13 mmol) were dissolved in 1.5 ml ethyl­enedi­amine in a Schlenk tube. The brown solution was stirred at ambient temperature for 1 h, then filtered and layered with 3.5 ml toluene. The solution was warmed in an oil bath to 323 K for 1 h, then stored at room temperature for crystallization. After 3 d, dark-brown plate-shaped crystals together with a small amount of elemental tin were found on the wall of the glass tube.

## Refinement   

Crystal data, data collection and structure refinement details are summarized in Table 1 ▸. All H atoms were included in calculated positions and treated as riding atoms with C—H = 0.99 Å and *U*_iso_(H) = 1.2*U*_eq_(C). The anisotropic displacement parameters of nine C atoms (C5, C8, C26, C29, C33, C45, C48, C50, C53) had to be restrained using the ISOR option (Sheldrick, 2015 ▸).

## Supplementary Material

Crystal structure: contains datablock(s) global, I. DOI: 10.1107/S2056989017000172/wm5352sup1.cif

Structure factors: contains datablock(s) I. DOI: 10.1107/S2056989017000172/wm5352Isup2.hkl

Supporting information file. DOI: 10.1107/S2056989017000172/wm5352Isup3.cdx

CCDC reference: 1525634

Additional supporting information:  crystallographic information; 3D view; checkCIF report

## Figures and Tables

**Table 1:** Experimental details

Crystal data	
Chemical formula	[Rb(C_18_H_36_N_2_O_6_)]_3_RbSn_9_
*M*_r_	2539.55
Crystal system, space group	Triclinic, *P* 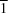
Temperature (K)	123
*a*, *b*, *c* (Å)	15.7287 (8), 16.153 (1), 20.2896 (8)
α, β, γ (°)	98.782 (4), 104.350 (4), 118.407 (6)
*V* (Å^3^)	4165.4 (4)
*Z*	2
Radiation type	Mo *K*α
μ (mm^−1^)	5.03
Crystal size (mm)	0.15 × 0.12 × 0.05

Data collection	
Diffractometer	Oxford Diffraction Xcalibur 3
Absorption correction	Multi-scan (*CrysAlis RED*; Oxford Diffraction, 2009 ▸)
*T*_min_, *T*_max_	0.832, 1.000
No. of measured, independent and observed [*I* > 2σ(*I*)] reflections	29013, 15902, 4724
*R*_int_	0.112
(sin θ/λ)_max_ (Å^−1^)	0.617

Refinement	
*R*[*F* ^2^ > 2σ(*F* ^2^)], *wR*(*F* ^2^), *S*	0.054, 0.094, 0.62
No. of reflections	15902
No. of parameters	823
No. of restraints	60
H-atom treatment	H-atom parameters constrained
Δρ_max_, Δρ_min_ (e Å^−3^)	2.85, −2.19

Computer programs: *CrysAlis CCD* and *CrysAlis RED* (Oxford Diffraction, 2009 ▸), *SHELXS97* (Sheldrick, 2008 ▸), *SHELXL2014/7* (Sheldrick, 2015 ▸), *DIAMOND* (Brandenburg, 2012 ▸) and *publCIF* (Westrip, 2010 ▸).

## supplementary crystallographic information

### Crystal data

**Table:**

[Rb(C_18_H_36_N_2_O_6_)]_3_RbSn_9_	*Z* = 2
*M**_r_* = 2539.55	*F*(000) = 2432
Triclinic, *P*1	*D*_x_ = 2.025 Mg m^−^^3^
*a* = 15.7287 (8) Å	Mo *K*α radiation, λ = 0.71073 Å
*b* = 16.153 (1) Å	Cell parameters from 2671 reflections
*c* = 20.2896 (8) Å	θ = 2.9–32.9°
α = 98.782 (4)°	µ = 5.03 mm^−^^1^
β = 104.350 (4)°	*T* = 123 K
γ = 118.407 (6)°	Plate, brown
*V* = 4165.4 (4) Å^3^	0.15 × 0.12 × 0.05 mm

### Data collection

**Table:**

Oxford Diffraction Xcalibur 3 diffractometer	15902 independent reflections
Radiation source: fine-focus sealed tube	4724 reflections with *I* > 2σ(*I*)
Graphite monochromator	*R*_int_ = 0.112
Detector resolution: 16.0238 pixels mm^-1^	θ_max_ = 26.0°, θ_min_ = 2.9°
ω and π scans	*h* = −17→19
Absorption correction: multi-scan (CrysAlisRED; Oxford Diffraction, 2009)	*k* = −19→19
*T*_min_ = 0.832, *T*_max_ = 1.000	*l* = −20→25
29013 measured reflections	

### Refinement

**Table:**

Refinement on *F*^2^	Primary atom site location: structure-invariant direct methods
Least-squares matrix: full	Secondary atom site location: difference Fourier map
*R*[*F*^2^ > 2σ(*F*^2^)] = 0.054	Hydrogen site location: inferred from neighbouring sites
*wR*(*F*^2^) = 0.094	H-atom parameters constrained
*S* = 0.62	*w* = 1/[σ^2^(*F*_o_^2^)] where *P* = (*F*_o_^2^ + 2*F*_c_^2^)/3
15902 reflections	(Δ/σ)_max_ = 0.001
823 parameters	Δρ_max_ = 2.85 e Å^−^^3^
60 restraints	Δρ_min_ = −2.19 e Å^−^^3^

### Special details

**Table:**

Geometry. All esds (except the esd in the dihedral angle between two l.s. planes) are estimated using the full covariance matrix. The cell esds are taken into account individually in the estimation of esds in distances, angles and torsion angles; correlations between esds in cell parameters are only used when they are defined by crystal symmetry. An approximate (isotropic) treatment of cell esds is used for estimating esds involving l.s. planes.

### Fractional atomic coordinates and isotropic or equivalent isotropic displacement parameters (Å^2^)

**Table:**

	*x*	*y*	*z*	*U*_iso_*/*U*_eq_	
Sn1	0.08357 (8)	0.16103 (7)	0.39428 (5)	0.0410 (3)	
Sn2	0.16023 (7)	0.25708 (7)	0.29264 (5)	0.0299 (3)	
Sn3	0.12360 (7)	0.07394 (7)	0.20224 (5)	0.0328 (3)	
Sn4	0.05539 (8)	−0.02245 (8)	0.30783 (5)	0.0416 (3)	
Sn5	−0.04374 (7)	0.21046 (7)	0.29624 (5)	0.0355 (3)	
Sn6	−0.01767 (8)	0.14432 (7)	0.15343 (5)	0.0343 (3)	
Sn7	−0.09899 (8)	−0.07686 (7)	0.16271 (5)	0.0386 (3)	
Sn8	−0.12916 (8)	−0.01594 (7)	0.30748 (5)	0.0419 (3)	
Sn9	−0.20912 (8)	0.02659 (8)	0.17892 (5)	0.0430 (3)	
Rb1	0.0000	0.0000	0.0000	0.0586 (7)	
Rb2	0.0000	0.0000	0.5000	0.1214 (13)	
Rb3	0.61092 (10)	0.39675 (10)	0.17403 (7)	0.0318 (4)	
N1	0.4557 (8)	0.2336 (7)	0.0330 (5)	0.028 (3)	
N2	0.7700 (9)	0.5604 (9)	0.3164 (6)	0.039 (3)	
O1	0.5734 (7)	0.4486 (7)	0.0473 (4)	0.039 (3)	
O2	0.7466 (7)	0.5961 (6)	0.1754 (5)	0.042 (3)	
O3	0.3993 (6)	0.2728 (6)	0.1544 (4)	0.031 (2)	
O4	0.5387 (8)	0.4516 (7)	0.2800 (5)	0.042 (3)	
O5	0.6478 (7)	0.2466 (7)	0.1151 (5)	0.043 (3)	
O6	0.7708 (7)	0.3809 (7)	0.2619 (5)	0.042 (3)	
C1	0.4354 (9)	0.2790 (9)	−0.0236 (6)	0.032 (4)	
H1A	0.3845	0.2967	−0.0185	0.039*	
H1B	0.4045	0.2296	−0.0716	0.039*	
C2	0.5381 (11)	0.3749 (11)	−0.0178 (7)	0.053 (5)	
H2A	0.5919	0.3596	−0.0185	0.063*	
H2B	0.5239	0.3988	−0.0588	0.063*	
C3	0.6589 (10)	0.5372 (10)	0.0478 (7)	0.035 (4)	
H3A	0.6363	0.5582	0.0070	0.042*	
H3B	0.7140	0.5259	0.0431	0.042*	
C4	0.7011 (11)	0.6183 (10)	0.1191 (7)	0.048 (4)	
H4A	0.7531	0.6833	0.1174	0.058*	
H4B	0.6435	0.6231	0.1266	0.058*	
C5	0.7826 (11)	0.6677 (10)	0.2414 (8)	0.057 (5)	
H5A	0.7228	0.6674	0.2494	0.068*	
H5B	0.8305	0.7343	0.2397	0.068*	
C6	0.8390 (11)	0.6477 (10)	0.3040 (8)	0.056 (5)	
H6A	0.8938	0.6406	0.2934	0.067*	
H6B	0.8732	0.7051	0.3480	0.067*	
C7	0.3610 (10)	0.1695 (10)	0.0410 (7)	0.047 (4)	
H7A	0.3701	0.1242	0.0656	0.057*	
H7B	0.3061	0.1284	−0.0072	0.057*	
C8	0.3253 (10)	0.2235 (10)	0.0829 (7)	0.041 (4)	
H8A	0.3200	0.2723	0.0609	0.049*	
H8B	0.2560	0.1758	0.0827	0.049*	
C9	0.3669 (10)	0.3255 (10)	0.1942 (7)	0.041 (4)	
H9A	0.2950	0.2797	0.1907	0.049*	
H9B	0.3684	0.3785	0.1746	0.049*	
C10	0.4416 (11)	0.3711 (11)	0.2729 (8)	0.047 (4)	
H10A	0.4119	0.3942	0.3034	0.056*	
H10B	0.4510	0.3202	0.2892	0.056*	
C11	0.6091 (12)	0.4900 (11)	0.3483 (7)	0.044 (4)	
H11A	0.6228	0.4393	0.3603	0.053*	
H11B	0.5797	0.5072	0.3820	0.053*	
C12	0.7103 (12)	0.5817 (11)	0.3572 (8)	0.062 (5)	
H12A	0.6951	0.6295	0.3405	0.074*	
H12B	0.7546	0.6136	0.4088	0.074*	
C13	0.4980 (10)	0.1743 (10)	0.0100 (7)	0.046 (5)	
H13A	0.4402	0.1099	−0.0260	0.055*	
H13B	0.5466	0.2099	−0.0132	0.055*	
C14	0.5522 (10)	0.1562 (10)	0.0701 (7)	0.039 (4)	
H14A	0.5078	0.1305	0.0982	0.047*	
H14B	0.5666	0.1057	0.0519	0.047*	
C15	0.7082 (11)	0.2306 (10)	0.1697 (7)	0.049 (5)	
H15A	0.7327	0.1911	0.1482	0.059*	
H15B	0.6652	0.1929	0.1954	0.059*	
C16	0.8002 (11)	0.3285 (10)	0.2217 (7)	0.041 (4)	
H16A	0.8482	0.3166	0.2546	0.049*	
H16B	0.8379	0.3692	0.1948	0.049*	
C17	0.8587 (11)	0.4697 (12)	0.3131 (7)	0.057 (5)	
H17A	0.8994	0.5125	0.2885	0.068*	
H17B	0.9039	0.4544	0.3454	0.068*	
C18	0.8242 (10)	0.5226 (11)	0.3557 (7)	0.043 (4)	
H18A	0.7784	0.4765	0.3762	0.052*	
H18B	0.8861	0.5786	0.3963	0.052*	
Rb4	0.63019 (10)	0.24191 (10)	0.48979 (7)	0.0307 (4)	
N3	0.4697 (9)	0.2786 (8)	0.5135 (6)	0.035 (3)	
N4	0.7945 (9)	0.2050 (8)	0.4680 (5)	0.034 (3)	
O7	0.4670 (7)	0.2095 (7)	0.3686 (4)	0.034 (2)	
O8	0.5935 (7)	0.1347 (6)	0.3462 (4)	0.038 (3)	
O9	0.6892 (7)	0.4381 (6)	0.5596 (4)	0.035 (3)	
O10	0.8322 (6)	0.4092 (6)	0.5085 (4)	0.036 (3)	
O11	0.5135 (7)	0.1394 (6)	0.5733 (5)	0.040 (3)	
O12	0.6924 (7)	0.1374 (6)	0.5723 (4)	0.032 (2)	
C19	0.4044 (9)	0.2752 (10)	0.4456 (6)	0.036 (4)	
H19A	0.4423	0.3388	0.4362	0.043*	
H19B	0.3400	0.2673	0.4502	0.043*	
C20	0.3762 (11)	0.1911 (10)	0.3831 (6)	0.039 (4)	
H20A	0.3454	0.1281	0.3944	0.047*	
H20B	0.3240	0.1846	0.3402	0.047*	
C21	0.4405 (9)	0.1419 (9)	0.3066 (6)	0.031 (4)	
H21A	0.3962	0.1480	0.2660	0.038*	
H21B	0.4006	0.0740	0.3093	0.038*	
C22	0.5395 (10)	0.1599 (9)	0.2945 (7)	0.034 (4)	
H22A	0.5212	0.1189	0.2457	0.040*	
H22B	0.5844	0.2306	0.2989	0.040*	
C23	0.6872 (10)	0.1476 (10)	0.3405 (7)	0.042 (4)	
H23A	0.7316	0.2160	0.3401	0.050*	
H23B	0.6701	0.1012	0.2944	0.050*	
C24	0.7461 (10)	0.1303 (9)	0.3996 (6)	0.031 (4)	
H24A	0.6984	0.0656	0.4040	0.037*	
H24B	0.8007	0.1254	0.3867	0.037*	
C25	0.5207 (11)	0.3747 (10)	0.5659 (7)	0.044 (4)	
H25A	0.5486	0.3706	0.6139	0.053*	
H25B	0.4684	0.3921	0.5665	0.053*	
C26	0.6076 (10)	0.4564 (10)	0.5531 (7)	0.037 (4)	
H26A	0.5818	0.4598	0.5045	0.045*	
H26B	0.6344	0.5208	0.5885	0.045*	
C27	0.7740 (12)	0.5146 (10)	0.5454 (7)	0.051 (5)	
H27A	0.7995	0.5809	0.5775	0.061*	
H27B	0.7492	0.5133	0.4952	0.061*	
C28	0.8625 (10)	0.4949 (11)	0.5584 (7)	0.043 (4)	
H28A	0.9243	0.5513	0.5556	0.051*	
H28B	0.8819	0.4894	0.6071	0.051*	
C29	0.9102 (10)	0.3877 (10)	0.5277 (6)	0.034 (4)	
H29A	0.9171	0.3751	0.5744	0.041*	
H29B	0.9778	0.4451	0.5326	0.041*	
C30	0.8821 (10)	0.2979 (10)	0.4711 (7)	0.043 (4)	
H30A	0.8657	0.3082	0.4238	0.052*	
H30B	0.9434	0.2921	0.4796	0.052*	
C31	0.4037 (10)	0.2025 (11)	0.5402 (7)	0.046 (4)	
H31A	0.3560	0.1396	0.4999	0.055*	
H31B	0.3606	0.2230	0.5571	0.055*	
C32	0.4622 (11)	0.1839 (11)	0.5995 (7)	0.039 (4)	
H32A	0.5145	0.2473	0.6385	0.046*	
H32B	0.4137	0.1390	0.6192	0.046*	
C33	0.5677 (11)	0.1164 (10)	0.6260 (7)	0.040 (4)	
H33A	0.5183	0.0688	0.6438	0.048*	
H33B	0.6199	0.1775	0.6670	0.048*	
C34	0.6205 (10)	0.0730 (10)	0.5979 (7)	0.035 (4)	
H34A	0.6571	0.0575	0.6365	0.042*	
H34B	0.5680	0.0101	0.5585	0.042*	
C35	0.7387 (12)	0.0917 (11)	0.5435 (7)	0.050 (5)	
H35A	0.6856	0.0341	0.5003	0.060*	
H35B	0.7677	0.0675	0.5793	0.060*	
C36	0.8262 (10)	0.1680 (10)	0.5237 (7)	0.036 (4)	
H36A	0.8774	0.2253	0.5675	0.044*	
H36B	0.8624	0.1371	0.5083	0.044*	
Rb5	0.76177 (10)	0.32954 (9)	0.86626 (7)	0.0300 (4)	
N5	0.7359 (9)	0.1513 (8)	0.9045 (6)	0.041 (3)	
N6	0.7851 (8)	0.5051 (8)	0.8284 (6)	0.031 (3)	
O13	0.8244 (7)	0.2143 (6)	0.7948 (5)	0.037 (3)	
O14	0.8891 (6)	0.4026 (6)	0.7829 (5)	0.033 (2)	
O15	0.5564 (6)	0.1735 (6)	0.8469 (4)	0.033 (2)	
O16	0.5787 (6)	0.3200 (6)	0.7796 (4)	0.034 (2)	
O17	0.8618 (7)	0.3529 (7)	1.0154 (4)	0.036 (3)	
O18	0.8401 (6)	0.5092 (6)	0.9782 (4)	0.032 (2)	
C37	0.7556 (11)	0.0977 (10)	0.8552 (7)	0.040 (4)	
H37A	0.6908	0.0551	0.8124	0.048*	
H37B	0.7703	0.0529	0.8773	0.048*	
C38	0.8434 (12)	0.1553 (10)	0.8296 (8)	0.049 (5)	
H38A	0.9099	0.1969	0.8712	0.058*	
H38B	0.8497	0.1082	0.7968	0.058*	
C39	0.8988 (11)	0.2580 (10)	0.7645 (7)	0.041 (4)	
H39A	0.8923	0.2060	0.7269	0.049*	
H39B	0.9694	0.2932	0.8019	0.049*	
C40	0.8837 (10)	0.3292 (10)	0.7326 (6)	0.035 (4)	
H40A	0.9377	0.3609	0.7122	0.041*	
H40B	0.8151	0.2926	0.6930	0.041*	
C41	0.8807 (10)	0.4729 (11)	0.7549 (7)	0.043 (4)	
H41A	0.8169	0.4388	0.7113	0.051*	
H41B	0.9410	0.5093	0.7409	0.051*	
C42	0.8769 (10)	0.5446 (10)	0.8074 (7)	0.039 (4)	
H42A	0.9398	0.5758	0.8513	0.047*	
H42B	0.8808	0.5977	0.7871	0.047*	
C43	0.6326 (10)	0.0921 (9)	0.9057 (6)	0.033 (4)	
H43A	0.6309	0.1235	0.9509	0.039*	
H43B	0.6187	0.0256	0.9059	0.039*	
C44	0.5489 (10)	0.0800 (10)	0.8449 (7)	0.036 (4)	
H44A	0.5527	0.0526	0.7994	0.043*	
H44B	0.4807	0.0320	0.8462	0.043*	
C45	0.4793 (10)	0.1614 (9)	0.7896 (7)	0.037 (4)	
H45A	0.4109	0.1143	0.7910	0.044*	
H45B	0.4833	0.1324	0.7446	0.044*	
C46	0.4873 (9)	0.2567 (9)	0.7893 (6)	0.030 (4)	
H46A	0.4264	0.2442	0.7502	0.036*	
H46B	0.4878	0.2882	0.8354	0.036*	
C47	0.5921 (10)	0.4135 (9)	0.7803 (6)	0.028 (4)	
H47A	0.5951	0.4468	0.8268	0.034*	
H47B	0.5330	0.4045	0.7415	0.034*	
C48	0.6920 (9)	0.4754 (9)	0.7695 (6)	0.027 (3)	
H48A	0.6904	0.4374	0.7255	0.032*	
H48B	0.6959	0.5358	0.7614	0.032*	
C49	0.8130 (10)	0.1830 (11)	0.9768 (8)	0.044 (4)	
H49A	0.7933	0.1245	0.9937	0.053*	
H49B	0.8815	0.2053	0.9727	0.053*	
C50	0.8251 (11)	0.2625 (10)	1.0317 (7)	0.044 (4)	
H50A	0.7572	0.2410	1.0366	0.052*	
H50B	0.8746	0.2740	1.0784	0.052*	
C51	0.8655 (11)	0.4296 (11)	1.0631 (7)	0.050 (5)	
H51A	0.9081	0.4417	1.1127	0.060*	
H51B	0.7942	0.4077	1.0612	0.060*	
C52	0.9099 (11)	0.5238 (10)	1.0454 (8)	0.049 (5)	
H52A	0.9197	0.5776	1.0833	0.058*	
H52B	0.9785	0.5432	1.0426	0.058*	
C53	0.8794 (10)	0.5975 (8)	0.9598 (6)	0.027 (3)	
H53A	0.9473	0.6172	0.9556	0.033*	
H53B	0.8899	0.6517	0.9978	0.033*	
C54	0.8060 (10)	0.5816 (10)	0.8918 (7)	0.040 (4)	
H54A	0.8325	0.6455	0.8815	0.048*	
H54B	0.7391	0.5630	0.8975	0.048*	

### Atomic displacement parameters (Å^2^)

**Table:**

	*U*^11^	*U*^22^	*U*^33^	*U*^12^	*U*^13^	*U*^23^
Sn1	0.0433 (7)	0.0303 (7)	0.0357 (6)	0.0122 (6)	0.0073 (5)	0.0139 (5)
Sn2	0.0253 (6)	0.0228 (6)	0.0318 (6)	0.0083 (5)	0.0088 (5)	0.0032 (5)
Sn3	0.0321 (7)	0.0283 (6)	0.0368 (6)	0.0170 (5)	0.0111 (5)	0.0071 (5)
Sn4	0.0408 (7)	0.0433 (7)	0.0478 (7)	0.0261 (6)	0.0149 (6)	0.0223 (6)
Sn5	0.0318 (7)	0.0330 (7)	0.0396 (7)	0.0195 (6)	0.0094 (5)	0.0050 (5)
Sn6	0.0375 (7)	0.0319 (6)	0.0299 (6)	0.0185 (6)	0.0077 (5)	0.0088 (5)
Sn7	0.0331 (7)	0.0208 (6)	0.0463 (7)	0.0102 (5)	0.0050 (5)	0.0025 (5)
Sn8	0.0334 (7)	0.0333 (7)	0.0536 (7)	0.0129 (6)	0.0171 (6)	0.0161 (6)
Sn9	0.0251 (6)	0.0373 (7)	0.0527 (7)	0.0137 (6)	0.0043 (5)	0.0069 (6)
Rb1	0.0697 (18)	0.0670 (17)	0.0312 (13)	0.0357 (15)	0.0161 (12)	0.0037 (12)
Rb2	0.095 (2)	0.167 (3)	0.126 (3)	0.064 (2)	0.052 (2)	0.112 (2)
Rb3	0.0273 (9)	0.0260 (8)	0.0358 (9)	0.0116 (7)	0.0087 (7)	0.0083 (7)
N1	0.033 (8)	0.014 (7)	0.030 (7)	0.010 (6)	0.013 (6)	−0.001 (6)
N2	0.022 (8)	0.045 (9)	0.036 (8)	0.008 (7)	0.012 (6)	0.010 (7)
O1	0.051 (7)	0.040 (7)	0.025 (6)	0.020 (6)	0.022 (5)	0.011 (5)
O2	0.037 (6)	0.035 (6)	0.031 (6)	0.008 (5)	0.004 (5)	0.010 (5)
O3	0.041 (6)	0.029 (6)	0.029 (6)	0.026 (5)	0.009 (5)	0.000 (5)
O4	0.047 (7)	0.053 (7)	0.026 (6)	0.027 (6)	0.008 (5)	0.017 (5)
O5	0.042 (7)	0.041 (7)	0.033 (6)	0.014 (6)	0.010 (5)	0.011 (5)
O6	0.033 (6)	0.056 (7)	0.034 (6)	0.029 (6)	0.006 (5)	0.003 (6)
C1	0.013 (8)	0.017 (8)	0.035 (9)	−0.009 (7)	0.001 (7)	0.003 (7)
C2	0.062 (12)	0.068 (13)	0.038 (10)	0.042 (11)	0.023 (9)	0.007 (10)
C3	0.048 (10)	0.034 (9)	0.045 (10)	0.028 (9)	0.023 (8)	0.034 (8)
C4	0.045 (11)	0.035 (10)	0.055 (11)	0.017 (9)	0.013 (9)	0.018 (9)
C5	0.058 (8)	0.030 (7)	0.064 (8)	0.012 (6)	0.016 (7)	0.017 (7)
C6	0.036 (11)	0.036 (10)	0.062 (12)	−0.003 (9)	0.012 (9)	0.017 (9)
C7	0.029 (10)	0.033 (10)	0.043 (10)	−0.003 (8)	0.008 (8)	−0.006 (8)
C8	0.023 (7)	0.037 (7)	0.047 (8)	0.011 (6)	0.006 (6)	0.007 (6)
C9	0.037 (10)	0.029 (9)	0.047 (10)	0.012 (8)	0.003 (8)	0.026 (8)
C10	0.055 (12)	0.051 (11)	0.070 (12)	0.044 (10)	0.045 (10)	0.021 (10)
C11	0.076 (13)	0.060 (12)	0.040 (10)	0.062 (11)	0.026 (10)	0.026 (9)
C12	0.053 (13)	0.045 (12)	0.063 (12)	0.021 (11)	−0.005 (10)	0.019 (10)
C13	0.020 (9)	0.030 (10)	0.054 (11)	−0.005 (8)	0.000 (8)	0.018 (9)
C14	0.046 (11)	0.036 (10)	0.037 (9)	0.020 (9)	0.027 (8)	0.007 (8)
C15	0.047 (11)	0.050 (11)	0.048 (10)	0.019 (9)	0.017 (9)	0.034 (9)
C16	0.044 (11)	0.037 (10)	0.032 (9)	0.011 (9)	0.018 (8)	0.018 (8)
C17	0.049 (12)	0.071 (13)	0.044 (11)	0.044 (11)	−0.012 (9)	0.010 (10)
C18	0.030 (10)	0.052 (11)	0.036 (10)	0.020 (9)	0.001 (8)	0.012 (9)
Rb4	0.0284 (9)	0.0277 (9)	0.0312 (8)	0.0145 (7)	0.0075 (7)	0.0053 (7)
N3	0.051 (9)	0.028 (7)	0.040 (8)	0.028 (7)	0.018 (7)	0.021 (7)
N4	0.058 (9)	0.025 (7)	0.020 (7)	0.018 (7)	0.022 (6)	0.012 (6)
O7	0.038 (7)	0.045 (7)	0.021 (6)	0.025 (6)	0.010 (5)	0.008 (5)
O8	0.053 (7)	0.037 (6)	0.036 (6)	0.033 (6)	0.014 (5)	0.015 (5)
O9	0.030 (6)	0.019 (6)	0.046 (6)	0.009 (5)	0.007 (5)	0.005 (5)
O10	0.025 (6)	0.019 (6)	0.032 (6)	0.001 (5)	−0.005 (5)	−0.006 (5)
O11	0.040 (7)	0.031 (6)	0.036 (6)	0.011 (5)	0.014 (5)	0.005 (5)
O12	0.033 (6)	0.022 (6)	0.038 (6)	0.007 (5)	0.017 (5)	0.016 (5)
C19	0.018 (8)	0.056 (11)	0.026 (8)	0.020 (8)	0.003 (7)	0.001 (8)
C20	0.045 (11)	0.044 (10)	0.023 (9)	0.030 (9)	−0.001 (8)	0.001 (8)
C21	0.027 (9)	0.022 (8)	0.024 (8)	0.000 (7)	0.011 (7)	−0.001 (7)
C22	0.029 (9)	0.019 (8)	0.046 (10)	0.013 (7)	0.008 (8)	0.003 (8)
C23	0.028 (10)	0.037 (10)	0.064 (11)	0.012 (8)	0.036 (9)	0.016 (9)
C24	0.035 (9)	0.031 (9)	0.035 (9)	0.020 (8)	0.015 (7)	0.019 (8)
C25	0.051 (11)	0.042 (10)	0.039 (9)	0.034 (10)	0.005 (8)	−0.004 (9)
C26	0.043 (8)	0.033 (7)	0.041 (7)	0.032 (6)	0.005 (6)	−0.001 (6)
C27	0.066 (13)	0.016 (9)	0.035 (9)	0.015 (9)	−0.014 (9)	0.003 (8)
C28	0.023 (9)	0.039 (10)	0.046 (10)	0.007 (8)	0.005 (8)	0.011 (9)
C29	0.041 (8)	0.034 (7)	0.024 (7)	0.012 (6)	0.017 (6)	0.020 (6)
C30	0.042 (10)	0.042 (10)	0.056 (11)	0.017 (9)	0.037 (9)	0.032 (9)
C31	0.031 (10)	0.056 (11)	0.039 (10)	0.019 (9)	0.011 (8)	0.000 (9)
C32	0.046 (10)	0.048 (11)	0.032 (9)	0.025 (9)	0.028 (8)	0.016 (8)
C33	0.041 (8)	0.044 (8)	0.019 (7)	0.012 (6)	0.016 (6)	0.005 (6)
C34	0.024 (9)	0.036 (10)	0.041 (9)	0.015 (8)	0.007 (7)	0.014 (8)
C35	0.082 (14)	0.049 (11)	0.039 (10)	0.046 (11)	0.027 (9)	0.017 (9)
C36	0.053 (11)	0.037 (10)	0.043 (10)	0.039 (9)	0.022 (8)	0.015 (8)
Rb5	0.0317 (9)	0.0231 (8)	0.0315 (8)	0.0122 (7)	0.0120 (7)	0.0075 (7)
N5	0.037 (8)	0.023 (7)	0.035 (8)	0.007 (7)	−0.001 (7)	−0.007 (6)
N6	0.029 (8)	0.032 (8)	0.045 (8)	0.024 (6)	0.014 (6)	0.021 (7)
O13	0.037 (6)	0.019 (6)	0.056 (7)	0.013 (5)	0.025 (5)	0.010 (5)
O14	0.039 (6)	0.024 (6)	0.048 (6)	0.028 (5)	0.011 (5)	0.010 (5)
O15	0.017 (5)	0.038 (6)	0.030 (6)	0.010 (5)	0.000 (4)	0.008 (5)
O16	0.027 (6)	0.028 (6)	0.042 (6)	0.015 (5)	0.011 (5)	0.007 (5)
O17	0.048 (7)	0.039 (6)	0.017 (5)	0.024 (6)	0.010 (5)	0.000 (5)
O18	0.041 (6)	0.019 (6)	0.026 (6)	0.013 (5)	0.004 (5)	0.004 (5)
C37	0.053 (11)	0.037 (10)	0.053 (11)	0.035 (9)	0.034 (9)	0.013 (9)
C38	0.073 (13)	0.026 (10)	0.057 (11)	0.038 (10)	0.022 (10)	0.001 (9)
C39	0.041 (10)	0.023 (9)	0.042 (10)	0.007 (8)	0.017 (8)	−0.001 (8)
C40	0.042 (10)	0.029 (9)	0.024 (8)	0.012 (8)	0.016 (7)	0.006 (7)
C41	0.040 (10)	0.065 (12)	0.042 (10)	0.041 (10)	0.016 (8)	0.018 (9)
C42	0.018 (9)	0.031 (9)	0.060 (11)	0.013 (8)	0.002 (8)	0.015 (8)
C43	0.042 (10)	0.022 (8)	0.014 (8)	0.002 (8)	0.007 (7)	0.009 (7)
C44	0.055 (11)	0.033 (9)	0.043 (10)	0.031 (9)	0.034 (9)	0.021 (8)
C45	0.031 (7)	0.017 (7)	0.052 (8)	0.002 (5)	0.023 (6)	0.007 (6)
C46	0.009 (8)	0.039 (10)	0.022 (8)	0.001 (7)	0.002 (6)	0.011 (7)
C47	0.035 (9)	0.016 (8)	0.029 (8)	0.014 (7)	0.007 (7)	0.003 (7)
C48	0.032 (7)	0.023 (7)	0.033 (7)	0.023 (6)	0.005 (5)	0.011 (5)
C49	0.039 (10)	0.061 (12)	0.076 (12)	0.040 (9)	0.042 (9)	0.050 (10)
C50	0.052 (8)	0.028 (7)	0.055 (8)	0.026 (6)	0.010 (6)	0.024 (6)
C51	0.029 (10)	0.084 (14)	0.025 (9)	0.023 (10)	0.007 (8)	0.022 (10)
C52	0.055 (11)	0.025 (9)	0.063 (12)	0.014 (9)	0.026 (10)	0.027 (9)
C53	0.036 (7)	0.010 (6)	0.029 (7)	0.006 (5)	0.017 (6)	0.003 (5)
C54	0.033 (10)	0.036 (10)	0.050 (10)	0.016 (8)	0.010 (8)	0.029 (9)

### Geometric parameters (Å, º)

**Table:**

Sn1—Sn2	2.9229 (14)	O11—C33	1.407 (14)
Sn1—Sn5	2.9387 (14)	O11—C32	1.448 (14)
Sn1—Sn4	2.9724 (15)	O12—C34	1.403 (14)
Sn1—Sn8	2.9850 (14)	O12—C35	1.416 (15)
Sn1—Rb2	3.5976 (10)	C19—C20	1.508 (15)
Sn1—Sn3	4.2360 (14)	C19—H19A	0.9900
Sn2—Sn3	2.9404 (14)	C19—H19B	0.9900
Sn2—Sn5	2.9493 (14)	C20—H20A	0.9900
Sn2—Sn6	2.9644 (13)	C20—H20B	0.9900
Sn2—Sn4	4.0981 (14)	C21—C22	1.533 (16)
Sn3—Sn7	2.9549 (14)	C21—H21A	0.9900
Sn3—Sn4	2.9552 (14)	C21—H21B	0.9900
Sn3—Sn6	2.9777 (14)	C22—H22A	0.9900
Sn3—Rb1	3.7925 (9)	C22—H22B	0.9900
Sn4—Sn8	2.9527 (15)	C23—C24	1.472 (15)
Sn4—Sn7	2.9957 (14)	C23—H23A	0.9900
Sn4—Rb2	4.2008 (11)	C23—H23B	0.9900
Sn5—Sn9	2.9453 (14)	C24—H24A	0.9900
Sn5—Sn6	3.1249 (14)	C24—H24B	0.9900
Sn5—Sn8	3.3110 (15)	C25—C26	1.496 (17)
Sn6—Sn9	2.9283 (15)	C25—H25A	0.9900
Sn6—Sn7	3.2267 (14)	C25—H25B	0.9900
Sn6—Rb1	3.7460 (10)	C26—H26A	0.9900
Sn7—Sn9	2.9613 (15)	C26—H26B	0.9900
Sn7—Sn8	3.1741 (15)	C27—C28	1.540 (17)
Sn7—Rb1	4.1017 (11)	C27—H27A	0.9900
Sn8—Sn9	2.9327 (14)	C27—H27B	0.9900
Sn8—Rb2	3.8357 (10)	C28—H28A	0.9900
Rb3—O3	2.830 (9)	C28—H28B	0.9900
Rb3—O1	2.834 (8)	C29—C30	1.499 (16)
Rb3—O6	2.847 (9)	C29—H29A	0.9900
Rb3—O4	2.877 (9)	C29—H29B	0.9900
Rb3—O2	2.886 (9)	C30—H30A	0.9900
Rb3—O5	2.907 (10)	C30—H30B	0.9900
Rb3—N1	3.034 (10)	C31—C32	1.487 (16)
Rb3—N2	3.063 (11)	C31—H31A	0.9900
N1—C7	1.422 (15)	C31—H31B	0.9900
N1—C13	1.483 (16)	C32—H32A	0.9900
N1—C1	1.502 (14)	C32—H32B	0.9900
N2—C6	1.420 (15)	C33—C34	1.474 (17)
N2—C18	1.430 (15)	C33—H33A	0.9900
N2—C12	1.514 (18)	C33—H33B	0.9900
O1—C2	1.413 (14)	C34—H34A	0.9900
O1—C3	1.420 (13)	C34—H34B	0.9900
O2—C4	1.382 (13)	C35—C36	1.545 (17)
O2—C5	1.407 (14)	C35—H35A	0.9900
O3—C8	1.423 (12)	C35—H35B	0.9900
O3—C9	1.429 (15)	C36—H36A	0.9900
O4—C11	1.362 (13)	C36—H36B	0.9900
O4—C10	1.411 (15)	Rb5—O13	2.858 (9)
O5—C15	1.411 (13)	Rb5—O15	2.875 (8)
O5—C14	1.432 (13)	Rb5—O18	2.880 (8)
O6—C16	1.392 (15)	Rb5—O14	2.882 (9)
O6—C17	1.412 (15)	Rb5—O16	2.897 (8)
C1—C2	1.579 (16)	Rb5—O17	2.915 (8)
C1—H1A	0.9900	Rb5—N6	2.929 (10)
C1—H1B	0.9900	Rb5—N5	2.963 (11)
C2—H2A	0.9900	N5—C37	1.411 (14)
C2—H2B	0.9900	N5—C43	1.451 (15)
C3—C4	1.539 (16)	N5—C49	1.477 (14)
C3—H3A	0.9900	N6—C48	1.446 (13)
C3—H3B	0.9900	N6—C42	1.479 (15)
C4—H4A	0.9900	N6—C54	1.487 (15)
C4—H4B	0.9900	O13—C38	1.367 (14)
C5—C6	1.532 (16)	O13—C39	1.396 (14)
C5—H5A	0.9900	O14—C41	1.389 (14)
C5—H5B	0.9900	O14—C40	1.396 (13)
C6—H6A	0.9900	O15—C45	1.366 (13)
C6—H6B	0.9900	O15—C44	1.452 (13)
C7—C8	1.508 (17)	O16—C46	1.398 (13)
C7—H7A	0.9900	O16—C47	1.419 (13)
C7—H7B	0.9900	O17—C51	1.419 (15)
C8—H8A	0.9900	O17—C50	1.420 (14)
C8—H8B	0.9900	O18—C53	1.412 (13)
C9—C10	1.537 (15)	O18—C52	1.427 (13)
C9—H9A	0.9900	C37—C38	1.524 (17)
C9—H9B	0.9900	C37—H37A	0.9900
C10—H10A	0.9900	C37—H37B	0.9900
C10—H10B	0.9900	C38—H38A	0.9900
C11—C12	1.514 (18)	C38—H38B	0.9900
C11—H11A	0.9900	C39—C40	1.486 (16)
C11—H11B	0.9900	C39—H39A	0.9900
C12—H12A	0.9900	C39—H39B	0.9900
C12—H12B	0.9900	C40—H40A	0.9900
C13—C14	1.460 (15)	C40—H40B	0.9900
C13—H13A	0.9900	C41—C42	1.483 (16)
C13—H13B	0.9900	C41—H41A	0.9900
C14—H14A	0.9900	C41—H41B	0.9900
C14—H14B	0.9900	C42—H42A	0.9900
C15—C16	1.504 (16)	C42—H42B	0.9900
C15—H15A	0.9900	C43—C44	1.472 (15)
C15—H15B	0.9900	C43—H43A	0.9900
C16—H16A	0.9900	C43—H43B	0.9900
C16—H16B	0.9900	C44—H44A	0.9900
C17—C18	1.492 (17)	C44—H44B	0.9900
C17—H17A	0.9900	C45—C46	1.484 (16)
C17—H17B	0.9900	C45—H45A	0.9900
C18—H18A	0.9900	C45—H45B	0.9900
C18—H18B	0.9900	C46—H46A	0.9900
Rb4—O9	2.845 (8)	C46—H46B	0.9900
Rb4—O7	2.848 (8)	C47—C48	1.504 (15)
Rb4—O12	2.869 (8)	C47—H47A	0.9900
Rb4—O10	2.909 (8)	C47—H47B	0.9900
Rb4—O11	2.914 (9)	C48—H48A	0.9900
Rb4—O8	2.914 (9)	C48—H48B	0.9900
Rb4—N3	2.983 (11)	C49—C50	1.466 (16)
Rb4—N4	3.030 (12)	C49—H49A	0.9900
N3—C25	1.440 (14)	C49—H49B	0.9900
N3—C31	1.465 (15)	C50—H50A	0.9900
N3—C19	1.476 (13)	C50—H50B	0.9900
N4—C24	1.433 (14)	C51—C52	1.488 (17)
N4—C36	1.447 (13)	C51—H51A	0.9900
N4—C30	1.455 (14)	C51—H51B	0.9900
O7—C21	1.355 (12)	C52—H52A	0.9900
O7—C20	1.431 (15)	C52—H52B	0.9900
O8—C22	1.415 (13)	C53—C54	1.457 (14)
O8—C23	1.425 (14)	C53—H53A	0.9900
O9—C26	1.428 (13)	C53—H53B	0.9900
O9—C27	1.456 (15)	C54—H54A	0.9900
O10—C28	1.370 (14)	C54—H54B	0.9900
O10—C29	1.411 (14)		
			
Sn2—Sn1—Sn5	60.42 (3)	O9—Rb4—O8	135.0 (3)
Sn2—Sn1—Sn4	88.07 (4)	O7—Rb4—O8	59.5 (3)
Sn5—Sn1—Sn4	106.08 (4)	O12—Rb4—O8	100.6 (2)
Sn2—Sn1—Sn8	105.93 (4)	O10—Rb4—O8	94.1 (3)
Sn5—Sn1—Sn8	67.96 (3)	O11—Rb4—O8	120.8 (2)
Sn4—Sn1—Sn8	59.42 (3)	O9—Rb4—N3	60.5 (3)
Sn2—Sn1—Rb2	166.42 (4)	O7—Rb4—N3	61.1 (3)
Sn5—Sn1—Rb2	126.75 (4)	O12—Rb4—N3	118.4 (3)
Sn4—Sn1—Rb2	78.86 (3)	O10—Rb4—N3	119.4 (3)
Sn8—Sn1—Rb2	70.58 (3)	O11—Rb4—N3	60.7 (3)
Sn2—Sn1—Sn3	43.91 (3)	O8—Rb4—N3	119.3 (3)
Sn5—Sn1—Sn3	79.76 (3)	O9—Rb4—N4	119.3 (3)
Sn4—Sn1—Sn3	44.23 (3)	O7—Rb4—N4	119.7 (3)
Sn8—Sn1—Sn3	79.25 (3)	O12—Rb4—N4	61.0 (3)
Rb2—Sn1—Sn3	123.08 (3)	O10—Rb4—N4	60.6 (3)
Sn1—Sn2—Sn3	92.51 (4)	O11—Rb4—N4	118.7 (3)
Sn1—Sn2—Sn5	60.06 (3)	O8—Rb4—N4	61.5 (3)
Sn3—Sn2—Sn5	106.10 (4)	N3—Rb4—N4	179.2 (3)
Sn1—Sn2—Sn6	105.01 (4)	C25—N3—C31	109.2 (11)
Sn3—Sn2—Sn6	60.57 (3)	C25—N3—C19	109.6 (10)
Sn5—Sn2—Sn6	63.80 (3)	C31—N3—C19	109.2 (11)
Sn1—Sn2—Sn4	46.46 (3)	C25—N3—Rb4	109.1 (8)
Sn3—Sn2—Sn4	46.11 (3)	C31—N3—Rb4	109.7 (8)
Sn5—Sn2—Sn4	82.44 (3)	C19—N3—Rb4	110.1 (8)
Sn6—Sn2—Sn4	81.83 (3)	C24—N4—C36	109.4 (10)
Sn2—Sn3—Sn7	105.68 (4)	C24—N4—C30	112.4 (10)
Sn2—Sn3—Sn4	88.07 (4)	C36—N4—C30	110.9 (11)
Sn7—Sn3—Sn4	60.91 (3)	C24—N4—Rb4	106.5 (8)
Sn2—Sn3—Sn6	60.12 (3)	C36—N4—Rb4	108.9 (8)
Sn7—Sn3—Sn6	65.90 (3)	C30—N4—Rb4	108.5 (8)
Sn4—Sn3—Sn6	104.91 (4)	C21—O7—C20	110.4 (9)
Sn2—Sn3—Rb1	119.31 (3)	C21—O7—Rb4	116.7 (8)
Sn7—Sn3—Rb1	73.70 (3)	C20—O7—Rb4	113.2 (7)
Sn4—Sn3—Rb1	132.27 (4)	C22—O8—C23	114.9 (11)
Sn6—Sn3—Rb1	65.91 (3)	C22—O8—Rb4	109.8 (7)
Sn2—Sn3—Sn1	43.58 (3)	C23—O8—Rb4	111.3 (7)
Sn7—Sn3—Sn1	80.14 (3)	C26—O9—C27	111.2 (10)
Sn4—Sn3—Sn1	44.55 (3)	C26—O9—Rb4	117.0 (7)
Sn6—Sn3—Sn1	78.59 (3)	C27—O9—Rb4	113.6 (7)
Rb1—Sn3—Sn1	142.08 (3)	C28—O10—C29	108.5 (10)
Sn8—Sn4—Sn3	105.57 (4)	C28—O10—Rb4	113.6 (8)
Sn8—Sn4—Sn1	60.50 (4)	C29—O10—Rb4	111.3 (7)
Sn3—Sn4—Sn1	91.22 (4)	C33—O11—C32	113.4 (10)
Sn8—Sn4—Sn7	64.49 (4)	C33—O11—Rb4	114.6 (8)
Sn3—Sn4—Sn7	59.54 (3)	C32—O11—Rb4	114.0 (8)
Sn1—Sn4—Sn7	104.90 (4)	C34—O12—C35	110.2 (10)
Sn8—Sn4—Sn2	82.24 (3)	C34—O12—Rb4	117.2 (7)
Sn3—Sn4—Sn2	45.81 (3)	C35—O12—Rb4	116.1 (8)
Sn1—Sn4—Sn2	45.47 (3)	N3—C19—C20	111.9 (11)
Sn7—Sn4—Sn2	81.35 (3)	N3—C19—H19A	109.2
Sn8—Sn4—Rb2	61.98 (3)	C20—C19—H19A	109.2
Sn3—Sn4—Rb2	148.37 (4)	N3—C19—H19B	109.2
Sn1—Sn4—Rb2	57.17 (3)	C20—C19—H19B	109.2
Sn7—Sn4—Rb2	125.08 (4)	H19A—C19—H19B	107.9
Sn2—Sn4—Rb2	102.56 (3)	O7—C20—C19	109.9 (11)
Sn1—Sn5—Sn9	107.87 (4)	O7—C20—H20A	109.7
Sn1—Sn5—Sn2	59.53 (3)	C19—C20—H20A	109.7
Sn9—Sn5—Sn2	109.20 (4)	O7—C20—H20B	109.7
Sn1—Sn5—Sn6	100.73 (4)	C19—C20—H20B	109.7
Sn9—Sn5—Sn6	57.60 (3)	H20A—C20—H20B	108.2
Sn2—Sn5—Sn6	58.34 (3)	O7—C21—C22	109.2 (10)
Sn1—Sn5—Sn8	56.68 (3)	O7—C21—H21A	109.8
Sn9—Sn5—Sn8	55.54 (3)	C22—C21—H21A	109.8
Sn2—Sn5—Sn8	97.60 (4)	O7—C21—H21B	109.8
Sn6—Sn5—Sn8	89.13 (4)	C22—C21—H21B	109.8
Sn9—Sn6—Sn2	109.25 (4)	H21A—C21—H21B	108.3
Sn9—Sn6—Sn3	108.76 (4)	O8—C22—C21	109.2 (11)
Sn2—Sn6—Sn3	59.32 (3)	O8—C22—H22A	109.8
Sn9—Sn6—Sn5	58.12 (3)	C21—C22—H22A	109.8
Sn2—Sn6—Sn5	57.87 (3)	O8—C22—H22B	109.8
Sn3—Sn6—Sn5	100.91 (4)	C21—C22—H22B	109.8
Sn9—Sn6—Sn7	57.27 (3)	H22A—C22—H22B	108.3
Sn2—Sn6—Sn7	98.65 (4)	O8—C23—C24	113.0 (11)
Sn3—Sn6—Sn7	56.71 (3)	O8—C23—H23A	109.0
Sn5—Sn6—Sn7	91.74 (4)	C24—C23—H23A	109.0
Sn9—Sn6—Rb1	112.40 (3)	O8—C23—H23B	109.0
Sn2—Sn6—Rb1	120.07 (4)	C24—C23—H23B	109.0
Sn3—Sn6—Rb1	67.56 (3)	H23A—C23—H23B	107.8
Sn5—Sn6—Rb1	163.05 (4)	N4—C24—C23	115.1 (11)
Sn7—Sn6—Rb1	71.63 (3)	N4—C24—H24A	108.5
Sn3—Sn7—Sn9	108.48 (4)	C23—C24—H24A	108.5
Sn3—Sn7—Sn4	59.55 (3)	N4—C24—H24B	108.5
Sn9—Sn7—Sn4	107.75 (4)	C23—C24—H24B	108.5
Sn3—Sn7—Sn8	100.23 (4)	H24A—C24—H24B	107.5
Sn9—Sn7—Sn8	56.98 (3)	N3—C25—C26	114.6 (12)
Sn4—Sn7—Sn8	57.10 (3)	N3—C25—H25A	108.6
Sn3—Sn7—Sn6	57.39 (3)	C26—C25—H25A	108.6
Sn9—Sn7—Sn6	56.29 (3)	N3—C25—H25B	108.6
Sn4—Sn7—Sn6	98.16 (4)	C26—C25—H25B	108.6
Sn8—Sn7—Sn6	89.80 (4)	H25A—C25—H25B	107.6
Sn3—Sn7—Rb1	62.56 (3)	O9—C26—C25	109.6 (11)
Sn9—Sn7—Rb1	102.78 (3)	O9—C26—H26A	109.8
Sn4—Sn7—Rb1	120.29 (4)	C25—C26—H26A	109.8
Sn8—Sn7—Rb1	149.75 (4)	O9—C26—H26B	109.8
Sn6—Sn7—Rb1	60.08 (2)	C25—C26—H26B	109.8
Sn9—Sn8—Sn4	109.68 (4)	H26A—C26—H26B	108.2
Sn9—Sn8—Sn1	106.97 (4)	O9—C27—C28	108.7 (10)
Sn4—Sn8—Sn1	60.08 (4)	O9—C27—H27A	109.9
Sn9—Sn8—Sn7	57.85 (3)	C28—C27—H27A	109.9
Sn4—Sn8—Sn7	58.41 (3)	O9—C27—H27B	109.9
Sn1—Sn8—Sn7	100.35 (4)	C28—C27—H27B	109.9
Sn9—Sn8—Sn5	55.90 (3)	H27A—C27—H27B	108.3
Sn4—Sn8—Sn5	97.73 (4)	O10—C28—C27	110.6 (11)
Sn1—Sn8—Sn5	55.36 (3)	O10—C28—H28A	109.5
Sn7—Sn8—Sn5	89.32 (4)	C27—C28—H28A	109.5
Sn9—Sn8—Rb2	164.75 (4)	O10—C28—H28B	109.5
Sn4—Sn8—Rb2	75.20 (3)	C27—C28—H28B	109.5
Sn1—Sn8—Rb2	62.20 (3)	H28A—C28—H28B	108.1
Sn7—Sn8—Rb2	132.01 (4)	O10—C29—C30	109.4 (11)
Sn5—Sn8—Rb2	109.68 (3)	O10—C29—H29A	109.8
Sn6—Sn9—Sn8	100.87 (4)	C30—C29—H29A	109.8
Sn6—Sn9—Sn5	64.28 (3)	O10—C29—H29B	109.8
Sn8—Sn9—Sn5	68.57 (3)	C30—C29—H29B	109.8
Sn6—Sn9—Sn7	66.44 (4)	H29A—C29—H29B	108.2
Sn8—Sn9—Sn7	65.17 (4)	N4—C30—C29	114.8 (11)
Sn5—Sn9—Sn7	101.06 (4)	N4—C30—H30A	108.6
Sn6—Rb1—Sn6^i^	180.0	C29—C30—H30A	108.6
Sn6—Rb1—Sn3	46.53 (2)	N4—C30—H30B	108.6
Sn6^i^—Rb1—Sn3	133.47 (2)	C29—C30—H30B	108.6
Sn6—Rb1—Sn3^i^	133.47 (2)	H30A—C30—H30B	107.5
Sn6^i^—Rb1—Sn3^i^	46.53 (2)	N3—C31—C32	114.2 (11)
Sn3—Rb1—Sn3^i^	180.0	N3—C31—H31A	108.7
Sn6—Rb1—Sn7	48.29 (2)	C32—C31—H31A	108.7
Sn6^i^—Rb1—Sn7	131.71 (2)	N3—C31—H31B	108.7
Sn3—Rb1—Sn7	43.75 (2)	C32—C31—H31B	108.7
Sn3^i^—Rb1—Sn7	136.26 (2)	H31A—C31—H31B	107.6
Sn6—Rb1—Sn7^i^	131.71 (2)	O11—C32—C31	110.0 (11)
Sn6^i^—Rb1—Sn7^i^	48.29 (2)	O11—C32—H32A	109.7
Sn3—Rb1—Sn7^i^	136.25 (2)	C31—C32—H32A	109.7
Sn3^i^—Rb1—Sn7^i^	43.75 (2)	O11—C32—H32B	109.7
Sn7—Rb1—Sn7^i^	180.0	C31—C32—H32B	109.7
Sn1—Rb2—Sn1^ii^	180.0	H32A—C32—H32B	108.2
Sn1—Rb2—Sn8^ii^	132.78 (2)	O11—C33—C34	111.9 (11)
Sn1^ii^—Rb2—Sn8^ii^	47.22 (2)	O11—C33—H33A	109.2
Sn1—Rb2—Sn8	47.22 (2)	C34—C33—H33A	109.2
Sn1^ii^—Rb2—Sn8	132.78 (2)	O11—C33—H33B	109.2
Sn8^ii^—Rb2—Sn8	180.0	C34—C33—H33B	109.2
Sn1—Rb2—Sn4^ii^	136.03 (2)	H33A—C33—H33B	107.9
Sn1^ii^—Rb2—Sn4^ii^	43.97 (2)	O12—C34—C33	112.0 (11)
Sn8^ii^—Rb2—Sn4^ii^	42.81 (2)	O12—C34—H34A	109.2
Sn8—Rb2—Sn4^ii^	137.19 (2)	C33—C34—H34A	109.2
Sn1—Rb2—Sn4	43.97 (2)	O12—C34—H34B	109.2
Sn1^ii^—Rb2—Sn4	136.03 (2)	C33—C34—H34B	109.2
Sn8^ii^—Rb2—Sn4	137.19 (2)	H34A—C34—H34B	107.9
Sn8—Rb2—Sn4	42.81 (2)	O12—C35—C36	109.2 (11)
Sn4^ii^—Rb2—Sn4	180.0	O12—C35—H35A	109.8
O3—Rb3—O1	96.0 (3)	C36—C35—H35A	109.8
O3—Rb3—O6	122.5 (3)	O12—C35—H35B	109.8
O1—Rb3—O6	136.2 (3)	C36—C35—H35B	109.8
O3—Rb3—O4	61.8 (3)	H35A—C35—H35B	108.3
O1—Rb3—O4	117.3 (3)	N4—C36—C35	116.3 (11)
O6—Rb3—O4	100.2 (3)	N4—C36—H36A	108.2
O3—Rb3—O2	137.2 (3)	C35—C36—H36A	108.2
O1—Rb3—O2	60.4 (3)	N4—C36—H36B	108.2
O6—Rb3—O2	95.8 (3)	C35—C36—H36B	108.2
O4—Rb3—O2	95.9 (3)	H36A—C36—H36B	107.4
O3—Rb3—O5	99.3 (2)	O13—Rb5—O15	99.4 (3)
O1—Rb3—O5	95.7 (3)	O13—Rb5—O18	143.0 (3)
O6—Rb3—O5	60.9 (3)	O15—Rb5—O18	113.2 (3)
O4—Rb3—O5	142.3 (3)	O13—Rb5—O14	58.0 (3)
O2—Rb3—O5	117.0 (3)	O15—Rb5—O14	139.6 (2)
O3—Rb3—N1	59.1 (3)	O18—Rb5—O14	101.6 (2)
O1—Rb3—N1	60.9 (3)	O13—Rb5—O16	115.9 (2)
O6—Rb3—N1	119.4 (3)	O15—Rb5—O16	58.8 (2)
O4—Rb3—N1	120.0 (3)	O18—Rb5—O16	96.4 (2)
O2—Rb3—N1	120.3 (3)	O14—Rb5—O16	98.7 (2)
O5—Rb3—N1	59.7 (3)	O13—Rb5—O17	100.7 (3)
O3—Rb3—N2	121.5 (3)	O15—Rb5—O17	96.8 (2)
O1—Rb3—N2	119.4 (3)	O18—Rb5—O17	59.8 (3)
O6—Rb3—N2	59.8 (3)	O14—Rb5—O17	118.8 (2)
O4—Rb3—N2	60.8 (3)	O16—Rb5—O17	137.8 (3)
O2—Rb3—N2	59.8 (3)	O13—Rb5—N6	119.2 (3)
O5—Rb3—N2	119.5 (3)	O15—Rb5—N6	117.9 (3)
N1—Rb3—N2	179.1 (3)	O18—Rb5—N6	60.5 (3)
C7—N1—C13	108.6 (10)	O14—Rb5—N6	62.6 (3)
C7—N1—C1	109.7 (11)	O16—Rb5—N6	60.9 (3)
C13—N1—C1	108.9 (11)	O17—Rb5—N6	118.8 (3)
C7—N1—Rb3	110.1 (7)	O13—Rb5—N5	61.3 (3)
C13—N1—Rb3	110.0 (8)	O15—Rb5—N5	61.5 (3)
C1—N1—Rb3	109.5 (7)	O18—Rb5—N5	119.5 (3)
C6—N2—C18	111.6 (11)	O14—Rb5—N5	117.9 (3)
C6—N2—C12	110.1 (13)	O16—Rb5—N5	118.5 (3)
C18—N2—C12	109.3 (11)	O17—Rb5—N5	61.4 (3)
C6—N2—Rb3	110.6 (8)	N6—Rb5—N5	179.4 (3)
C18—N2—Rb3	107.5 (8)	C37—N5—C43	112.2 (11)
C12—N2—Rb3	107.5 (8)	C37—N5—C49	108.2 (12)
C2—O1—C3	107.1 (10)	C43—N5—C49	108.6 (11)
C2—O1—Rb3	116.2 (8)	C37—N5—Rb5	108.9 (9)
C3—O1—Rb3	114.4 (7)	C43—N5—Rb5	109.9 (8)
C4—O2—C5	111.0 (11)	C49—N5—Rb5	109.1 (8)
C4—O2—Rb3	112.0 (7)	C48—N6—C42	111.3 (10)
C5—O2—Rb3	112.5 (7)	C48—N6—C54	111.6 (10)
C8—O3—C9	108.4 (10)	C42—N6—C54	108.7 (10)
C8—O3—Rb3	117.7 (7)	C48—N6—Rb5	108.9 (7)
C9—O3—Rb3	111.0 (7)	C42—N6—Rb5	105.8 (7)
C11—O4—C10	111.6 (11)	C54—N6—Rb5	110.4 (7)
C11—O4—Rb3	113.3 (8)	C38—O13—C39	110.0 (11)
C10—O4—Rb3	110.7 (7)	C38—O13—Rb5	115.5 (8)
C15—O5—C14	111.8 (10)	C39—O13—Rb5	119.9 (7)
C15—O5—Rb3	111.5 (8)	C41—O14—C40	112.9 (10)
C14—O5—Rb3	111.2 (8)	C41—O14—Rb5	114.4 (8)
C16—O6—C17	111.1 (11)	C40—O14—Rb5	114.7 (7)
C16—O6—Rb3	112.1 (8)	C45—O15—C44	111.1 (10)
C17—O6—Rb3	116.7 (8)	C45—O15—Rb5	114.1 (7)
N1—C1—C2	111.9 (10)	C44—O15—Rb5	112.4 (7)
N1—C1—H1A	109.2	C46—O16—C47	112.5 (10)
C2—C1—H1A	109.2	C46—O16—Rb5	114.6 (7)
N1—C1—H1B	109.2	C47—O16—Rb5	114.8 (7)
C2—C1—H1B	109.2	C51—O17—C50	115.1 (11)
H1A—C1—H1B	107.9	C51—O17—Rb5	111.3 (7)
O1—C2—C1	108.0 (11)	C50—O17—Rb5	114.3 (7)
O1—C2—H2A	110.1	C53—O18—C52	110.3 (9)
C1—C2—H2A	110.1	C53—O18—Rb5	116.3 (7)
O1—C2—H2B	110.1	C52—O18—Rb5	115.4 (7)
C1—C2—H2B	110.1	N5—C37—C38	118.6 (12)
H2A—C2—H2B	108.4	N5—C37—H37A	107.7
O1—C3—C4	108.4 (11)	C38—C37—H37A	107.7
O1—C3—H3A	110.0	N5—C37—H37B	107.7
C4—C3—H3A	110.0	C38—C37—H37B	107.7
O1—C3—H3B	110.0	H37A—C37—H37B	107.1
C4—C3—H3B	110.0	O13—C38—C37	110.4 (12)
H3A—C3—H3B	108.4	O13—C38—H38A	109.6
O2—C4—C3	110.1 (11)	C37—C38—H38A	109.6
O2—C4—H4A	109.6	O13—C38—H38B	109.6
C3—C4—H4A	109.6	C37—C38—H38B	109.6
O2—C4—H4B	109.6	H38A—C38—H38B	108.1
C3—C4—H4B	109.6	O13—C39—C40	109.7 (12)
H4A—C4—H4B	108.2	O13—C39—H39A	109.7
O2—C5—C6	111.6 (12)	C40—C39—H39A	109.7
O2—C5—H5A	109.3	O13—C39—H39B	109.7
C6—C5—H5A	109.3	C40—C39—H39B	109.7
O2—C5—H5B	109.3	H39A—C39—H39B	108.2
C6—C5—H5B	109.3	O14—C40—C39	112.2 (11)
H5A—C5—H5B	108.0	O14—C40—H40A	109.2
N2—C6—C5	111.9 (12)	C39—C40—H40A	109.2
N2—C6—H6A	109.2	O14—C40—H40B	109.2
C5—C6—H6A	109.2	C39—C40—H40B	109.2
N2—C6—H6B	109.2	H40A—C40—H40B	107.9
C5—C6—H6B	109.2	O14—C41—C42	111.9 (11)
H6A—C6—H6B	107.9	O14—C41—H41A	109.2
N1—C7—C8	113.7 (11)	C42—C41—H41A	109.2
N1—C7—H7A	108.8	O14—C41—H41B	109.2
C8—C7—H7A	108.8	C42—C41—H41B	109.2
N1—C7—H7B	108.8	H41A—C41—H41B	107.9
C8—C7—H7B	108.8	N6—C42—C41	116.9 (11)
H7A—C7—H7B	107.7	N6—C42—H42A	108.1
O3—C8—C7	108.0 (11)	C41—C42—H42A	108.1
O3—C8—H8A	110.1	N6—C42—H42B	108.1
C7—C8—H8A	110.1	C41—C42—H42B	108.1
O3—C8—H8B	110.1	H42A—C42—H42B	107.3
C7—C8—H8B	110.1	N5—C43—C44	114.3 (10)
H8A—C8—H8B	108.4	N5—C43—H43A	108.7
O3—C9—C10	108.2 (11)	C44—C43—H43A	108.7
O3—C9—H9A	110.1	N5—C43—H43B	108.7
C10—C9—H9A	110.1	C44—C43—H43B	108.7
O3—C9—H9B	110.1	H43A—C43—H43B	107.6
C10—C9—H9B	110.1	O15—C44—C43	111.7 (11)
H9A—C9—H9B	108.4	O15—C44—H44A	109.3
O4—C10—C9	109.9 (11)	C43—C44—H44A	109.3
O4—C10—H10A	109.7	O15—C44—H44B	109.3
C9—C10—H10A	109.7	C43—C44—H44B	109.3
O4—C10—H10B	109.7	H44A—C44—H44B	107.9
C9—C10—H10B	109.7	O15—C45—C46	112.0 (11)
H10A—C10—H10B	108.2	O15—C45—H45A	109.2
O4—C11—C12	111.4 (12)	C46—C45—H45A	109.2
O4—C11—H11A	109.3	O15—C45—H45B	109.2
C12—C11—H11A	109.3	C46—C45—H45B	109.2
O4—C11—H11B	109.3	H45A—C45—H45B	107.9
C12—C11—H11B	109.3	O16—C46—C45	109.7 (11)
H11A—C11—H11B	108.0	O16—C46—H46A	109.7
N2—C12—C11	113.5 (13)	C45—C46—H46A	109.7
N2—C12—H12A	108.9	O16—C46—H46B	109.7
C11—C12—H12A	108.9	C45—C46—H46B	109.7
N2—C12—H12B	108.9	H46A—C46—H46B	108.2
C11—C12—H12B	108.9	O16—C47—C48	108.1 (10)
H12A—C12—H12B	107.7	O16—C47—H47A	110.1
C14—C13—N1	112.1 (12)	C48—C47—H47A	110.1
C14—C13—H13A	109.2	O16—C47—H47B	110.1
N1—C13—H13A	109.2	C48—C47—H47B	110.1
C14—C13—H13B	109.2	H47A—C47—H47B	108.4
N1—C13—H13B	109.2	N6—C48—C47	114.7 (10)
H13A—C13—H13B	107.9	N6—C48—H48A	108.6
O5—C14—C13	109.9 (11)	C47—C48—H48A	108.6
O5—C14—H14A	109.7	N6—C48—H48B	108.6
C13—C14—H14A	109.7	C47—C48—H48B	108.6
O5—C14—H14B	109.7	H48A—C48—H48B	107.6
C13—C14—H14B	109.7	C50—C49—N5	116.2 (11)
H14A—C14—H14B	108.2	C50—C49—H49A	108.2
O5—C15—C16	110.0 (11)	N5—C49—H49A	108.2
O5—C15—H15A	109.7	C50—C49—H49B	108.2
C16—C15—H15A	109.7	N5—C49—H49B	108.2
O5—C15—H15B	109.7	H49A—C49—H49B	107.4
C16—C15—H15B	109.7	O17—C50—C49	112.7 (12)
H15A—C15—H15B	108.2	O17—C50—H50A	109.1
O6—C16—C15	112.2 (13)	C49—C50—H50A	109.1
O6—C16—H16A	109.2	O17—C50—H50B	109.1
C15—C16—H16A	109.2	C49—C50—H50B	109.1
O6—C16—H16B	109.2	H50A—C50—H50B	107.8
C15—C16—H16B	109.2	O17—C51—C52	112.4 (12)
H16A—C16—H16B	107.9	O17—C51—H51A	109.1
O6—C17—C18	109.4 (12)	C52—C51—H51A	109.1
O6—C17—H17A	109.8	O17—C51—H51B	109.1
C18—C17—H17A	109.8	C52—C51—H51B	109.1
O6—C17—H17B	109.8	H51A—C51—H51B	107.9
C18—C17—H17B	109.8	O18—C52—C51	108.6 (11)
H17A—C17—H17B	108.2	O18—C52—H52A	110.0
N2—C18—C17	114.9 (12)	C51—C52—H52A	110.0
N2—C18—H18A	108.5	O18—C52—H52B	110.0
C17—C18—H18A	108.5	C51—C52—H52B	110.0
N2—C18—H18B	108.5	H52A—C52—H52B	108.3
C17—C18—H18B	108.5	O18—C53—C54	109.4 (10)
H18A—C18—H18B	107.5	O18—C53—H53A	109.8
O9—Rb4—O7	94.6 (3)	C54—C53—H53A	109.8
O9—Rb4—O12	119.1 (2)	O18—C53—H53B	109.8
O7—Rb4—O12	141.6 (2)	C54—C53—H53B	109.8
O9—Rb4—O10	60.3 (3)	H53A—C53—H53B	108.2
O7—Rb4—O10	112.6 (2)	C53—C54—N6	116.3 (11)
O12—Rb4—O10	100.5 (2)	C53—C54—H54A	108.2
O9—Rb4—O11	98.7 (3)	N6—C54—H54A	108.2
O7—Rb4—O11	101.1 (3)	C53—C54—H54B	108.2
O12—Rb4—O11	58.7 (3)	N6—C54—H54B	108.2
O10—Rb4—O11	140.8 (2)	H54A—C54—H54B	107.4

Symmetry codes: (i) −*x*, −*y*, −*z*; (ii) −*x*, −*y*, −*z*+1.
